# DNA Methylation of PGC-1α Is Associated With Elevated mtDNA Copy Number and Altered Urinary Metabolites in Autism Spectrum Disorder

**DOI:** 10.3389/fcell.2021.696428

**Published:** 2021-07-26

**Authors:** Sophia Bam, Erin Buchanan, Caitlyn Mahony, Colleen O’Ryan

**Affiliations:** Department of Molecular and Cell Biology, University of Cape Town, Cape Town, South Africa

**Keywords:** Autism Spectrum Disorder, methylation, PGC-1α, mtDNA copy number, metabolomics, mitochondrial dysfunction

## Abstract

Autism spectrum disorder (ASD) is a complex disorder that is underpinned by numerous dysregulated biological pathways, including pathways that affect mitochondrial function. Epigenetic mechanisms contribute to this dysregulation and DNA methylation is an important factor in the etiology of ASD. We measured DNA methylation of peroxisome proliferator-activated receptor-gamma coactivator-1 alpha (*PGC-1*α), as well as five genes involved in regulating mitochondrial homeostasis to examine mitochondrial dysfunction in an ASD cohort of South African children. Using targeted Next Generation bisulfite sequencing, we found differential methylation (*p* < 0.05) at six key genes converging on mitochondrial biogenesis, fission and fusion in ASD, namely *PGC-1*α, *STOML2*, *MFN2*, *FIS1*, *OPA1*, and *GABPA. PGC-1*α, the transcriptional regulator of biogenesis, was significantly hypermethylated at eight CpG sites in the gene promoter, one of which contained a putative binding site for CAMP response binding element 1 (CREB1) (*p* = 1 × 10^–6^). Mitochondrial DNA (mtDNA) copy number, a marker of mitochondrial function, was elevated (*p* = 0.002) in ASD compared to controls and correlated significantly with DNA methylation at the *PGC-1*α promoter and there was a positive correlation between methylation at *PGC-1*α CpG#1 and mtDNA copy number (Spearman’s *r* = 0.2, *n* = 49, *p* = 0.04) in ASD. Furthermore, DNA methylation at *PGC-1*α CpG#1 and mtDNA copy number correlated significantly (*p* < 0.05) with levels of urinary organic acids associated with mitochondrial dysfunction, oxidative stress, and neuroendocrinology. Our data show differential methylation in ASD at six key genes converging on *PGC-1*α-dependent regulation of mitochondrial biogenesis and function. We demonstrate that methylation at the *PGC-1*α promoter is associated with elevated mtDNA copy number and metabolomic evidence of mitochondrial dysfunction in ASD. This highlights an unexplored role for DNA methylation in regulating specific pathways involved in mitochondrial biogenesis, fission and fusion contributing to mitochondrial dysfunction in ASD.

## Introduction

Autism spectrum disorder (ASD) is defined by the presence of behavioral traits (Lord et al., 2020) despite being a highly heritable neurodevelopmental disorder (Sandin et al., 2017). ASD is characterized by deficits in social communication and restrictive, repetitive behaviors (American Psychiatric Association [APA], 2013). ASD is a complex disorder that affects the central nervous system as well as other organ systems, implying the dysregulation of pleiotropic biological and developmental pathways. ASD is underpinned by a heterogeneous genetic architecture that includes rare *de novo* genetic variations and low risk, common single nucleotide mutations (Gaugler et al., 2014; Feliciano et al., 2019). There is increasing evidence for the role of DNA methylation in modulating ASD phenotypes. This is evident from discordant identical ASD twin studies (Wong et al., 2014), studies using brain tissue from individuals with ASD (Ladd-Acosta et al., 2014; Nardone et al., 2014), with recent reviews collating numerous reports on ASD epigenetics (Rylaarsdam and Guemez-Gamboa, 2019; Tremblay and Jiang, 2019; Wiśniowiecka-Kowalnik and Nowakowska, 2019; Yoon et al., 2020).

Given the varied phenotypes and co-morbidities observed in individuals with ASD, numerous and diverse biological pathways have been implicated in ASD etiology. These include gene regulatory-, signaling-, synaptic-, and mitochondrial-pathways (Frye and Rossignol, 2011; Voineagu, 2012; Ayhan and Konopka, 2019; Kumar et al., 2019). Mitochondrial dysfunction is emerging as a key contributor to ASD etiology; deficiencies in oxidative phosphorylation (OXPHOS) can decrease the production of ATP, which is an essential requirement for brain function and neurodevelopment (Valenti et al., 2014). The observation that congenital errors of mitochondrial metabolism contribute to >5% of ASD cases (Manzi et al., 2008) first implied a role for mitochondrial dysfunction in ASD. This has since been supported by clinical (Oliveira et al., 2005), biochemical (Melnyk et al., 2012; Tang et al., 2013), molecular (Dhillon et al., 2011) and more recently, epigenetic data (Stathopoulos et al., 2020). Citrigno et al. (2020) comprehensively reviewed the recent experimental data that support a role for mitochondrial dysfunction in ASD.

Mitochondrial homeostasis is dynamic and is regulated by interdependent pathways that govern mitochondrial biogenesis, mitophagy, mitochondrial fission and fusion (Ventura-Clapier et al., 2008; Ploumi et al., 2017). These dynamic mechanisms enable cellular adaptation to change energy demands, nutrient availability, and oxidative stress by regulating mitochondrial DNA (mtDNA) copy number (Gaziev et al., 2014; Santos et al., 2014). Abnormal or fluctuating levels of mtDNA copy number is a marker of mitochondrial dysfunction (Malik and Czajka, 2013; Sun et al., 2019). Oxidative stress and mitochondrial dysfunction can lead to increased mtDNA copy number as a compensatory mechanism to maintain cellular homeostasis (Lee et al., 2000; Michel et al., 2012; Petersen et al., 2014; Kim et al., 2019). An essential transcriptional regulator of mitochondrial homeostasis is peroxisome proliferator-activated receptor-gamma coactivator-1 alpha (*PGC-1*α) which regulates fatty acid β-oxidation, OXPHOS, gluconeogenesis and antioxidant defense responses (Barone et al., 2019). *PGC-1*α catalyzes mitochondrial biogenesis by upregulating nuclear respiratory factors 1 (*Nrf-1*) and 2 (*Nrf-2*), also known as GA binding protein transcription factor subunit alpha (GABPA), which promotes the transcription of mitochondrial transcription factors A (*TFAM*) and B2 (*TFB2M*) (Ventura-Clapier et al., 2008; Ploumi et al., 2017). Importantly, numerous studies report a correlation between DNA methylation of the *PGC-1*α promoter and *PGC-1*α transcription, mtDNA copy number and metabolic disease (Barrès et al., 2009; Sookoian et al., 2010; Heinonen et al., 2015; Kresovich et al., 2018), suggesting that DNA methylation regulates *PGC-1*α-driven mitochondrial biogenesis.

Similarly, mitochondrial fission and fusion maintain mitochondrial homeostasis by limiting intracellular reactive oxygen species (ROS; Karbowski and Youle, 2003). Mitochondrial fusion occurs in response to mild oxidative stress and is mediated by mitofusins 1 and 2 (*MFN1* and *MFN2*) and optic atrophy 1 (*OPA1*) (Chen et al., 2003; Duvezin-Caubet et al., 2006; Ishihara et al., 2006). These proteins work in conjunction with accessory proteins, such as stomatin-like protein 2 (*STOML2*), which maintains the long isoforms of *OPA1* needed to fuse the inner mitochondrial membranes (Tondera et al., 2009). Fission occurs during severe oxidative stress and separates damaged mitochondrial components from the healthy mitochondrial network. Fission is mediated by dynamin-related protein 1 (*DRP1*) which works with mitochondrial fission protein 1 (*FIS1*) to divide the outer mitochondrial membrane (Smirnova et al., 2001; Stojanovski et al., 2004).

Recently, genes involved in mitochondrial biogenesis, fission and fusion have been implicated in neuropathology. *PGC-1*α is reported to play an important role in excitatory neurotransmitter signaling, neuroprotection, neuroinflammation and neurogenesis and has been implicated in bipolar disorder, Parkinson’s disease, Huntington’s disease, schizophrenia, and Alzheimer’s disease (Bu et al., 2017). In addition, recent studies have implicated *PGC-1*α (Barone et al., 2019), fusion and fission genes (Carrasco et al., 2019; Pecorelli et al., 2020) in ASD etiology (Barone et al., 2019), while *STOML2* was the most significantly differentially methylated (DM) gene in a South African ASD population (Stathopoulos et al., 2020).

Although there has been a massive increase in ASD genetic data, it has been primarily from ASD samples from Northern hemisphere countries. Our recent study identified DM genes in a South African cohort that were enriched for mitochondrial pathways, supporting a role for epigenetic dysregulation of energy metabolism in ASD. In view of our previous data (Stathopoulos et al., 2020) and the evidence from literature implicating both differential DNA methylation and mitochondrial dysfunction in ASD, we examined the relationship between these two processes in South African children with ASD. First, we determined whether *PGC-1*α was DM between ASD and matched controls from a South African population. Secondly, we measured DNA methylation of five additional genes that are central to mitochondrial biogenesis (GABPA), fission (*FIS1*) and fusion (*MFN2*, *STOML2*, and *OPA1*). Subsequently, we addressed whether DNA methylation modified mitochondrial function, which was measured using mtDNA copy number and urinary metabolomics. Overall, our aim was to test the hypothesis that DNA methylation of mitochondrial biogenesis genes changes mitochondrial function, thereby contributing to the etiology of ASD.

## Materials and Methods

### Participants and Sample Collection

Given that South Africa has no centralized ASD network with an associated repository of DNA, RNA and/or ASD phenotypes, we recruited children with ASD and age- and gender-matched typically developing controls (Stathopoulos et al., 2020). We recruited both male and female participants (6–17 years), but only pre-pubertal boys (6–12 years old) were used given that we recruited fewer than five females. We screened 145 participants (93 ASD; 52 controls) from three demographic groups: African-, European-, and Mixed-ancestry. An Autism Diagnostic Observation Schedule, Second Edition (ADOS-2) assessment was used to phenotype the ASD group, as well as to ensure the absence of ASD traits in the control group. The study protocol was approved by the University of Cape Town, as well as the Western Cape Government approval to recruit participants at schools. Buccal cells were collected from participants for DNA extraction; this is a minimally invasive collection method suited for DNA methylation studies (Lowe et al., 2013; Berko et al., 2014; Smith et al., 2015). Participants who did not consent to providing buccal samples or those samples which yielded poor DNA quality were excluded from the study. Urine samples were collected for organic acids extraction for metabolomic analysis using gas chromatography–mass spectrometry (GC–MS).

### Targeted Next-Generation Bisulfite Sequencing

DNA was extracted from buccal cells as previously described (Stathopoulos et al., 2020). DNA methylation was quantified using targeted Next-Generation Bisulfite Sequencing (tNGBS). After excluding participants who would not provide a DNA sample or whose buccal cells yielded DNA of poor quality, we proceeded to DNA methylation quantification using tNGBS. First, we quantified methylation for *PGC-1*α and *STOML2*, both of which were DM in our previous discovery cohort, in a larger validation cohort of ASD (*n* = 55) and controls (*n* = 44). We also quantified methylation for additional genes required for mitochondrial fusion and fission *FIS1, MFN2, OPA1*, and GABPA in a subset of samples (ASD, *n* = 22; controls *n* = 22). The tNGBS was completed by EpigenDx, Inc. (MA, United States) who designed a total of 32 tNGBS assays to analyze 171 CpG sites across six genes. tNGBS assays were designed by (i) obtaining and annotating each gene sequence using the Ensembl genome browser, (ii) re-evaluating target EPIC array probe sequences against the UCSC genome browser to identify LINE, SINE, LTR elements and other DNA repeat sequences; (iii) excluding sequences containing repetitive elements, low sequences complexity, high thymidine content and overall CpG density, and (iv) identifying assays that passed PCR optimization. This process was used to designed seven assays that analyzed 26 CpG sites for *PGC-1*α, eight assays that analyzed 45 CpG sites for *STOML2*, four assays covering 30 CpG sites for *FIS1*, five assays covering 26 CpG sites for *MFN2*, five assays covering 25 CpG sites for *OPA1* and three assays that analyzed 19 CpG sites for GABPA. Methylation levels were calculated by dividing the number of methylated reads by the number of total reads. Unpaired two-tailed *t*-tests with unequal variance were used to determine the significantly DM CpG sites between ASD and control (*p* < 0.05).

### Mitochondrial DNA Copy Number and Deletion

Mitochondrial DNA copy number and mitochondrial deletions were measured using multiplex real-time quantitative polymerase chain reaction (RT-qPCR) in 108 participants (ASD *n* = 68; controls *n* = 40). Mitochondrial genes mitochondrially encoded NADH: ubiquinone oxidoreductase core subunit 1 (*MT-ND1*) and mitochondrially encoded NADH: ubiquinone oxidoreductase core subunit 4 (*MT-ND4*), were amplified by RT-qPCR and normalized to the nuclear gene, beta-2-microglobulin (*B2M*), in the same PCR reaction. The probes were coupled to non-fluorescent quenchers (BHQ^®^, LGC BioSearch) and both the primers and probes used were previously reported by Grady et al. (2014). Each DNA sample (20 ng/μl) was amplified in triplicate, with standard curves set for each gene using equimolar pooled DNA from ASD and controls in a 10-fold dilution series. A two-step thermal profile was used with denaturation at 95°C for 10 min, followed by 40 cycles of 10 s at 95°C, 30 s at 60°C on the Rotor-Gene Q 6-plex (QIAGEN). The raw data was checked for the presence of outliers that were the result of poor qPCR detection. Samples with a triplicate threshold cycle Ct standard deviation greater than 1 were removed before analysis. A total of 99 samples (ASD *n* = 59; controls *n* = 40) were used in subsequent analyzes. Mitochondrial copy number was calculated using the equation 2^−ΔΔCt^, where ΔCt = Ct (*MT-ND1*) - Ct (*B2M*). Mitochondrial deletion was calculated using the equation 2^−ΔΔCt^, where ΔCt = Ct (*MT-ND4*) - Ct (*MT-ND1*). Significance was determined using a two-tailed unpaired *t*-test *p* < 0.05 was considered statistically significant.

### Metabolomic Correlation Analysis

We examined the correlation between *PGC-1*α methylation, mtDNA copy number and levels of urinary organic acids associated with mitochondrial dysfunction. Urinary organic acids for 35 participants (ASD *n* = 21 and controls *n* = 13) had been previously extracted and quantified by GC–MS (Stathopoulos et al., 2020) to screen for a panel of metabolites that were indicative of mitochondrial respiratory disease in South African children (Reinecke et al., 2012). The GC–MS data had been analyzed and deconvoluted using a standard metabolomics-based data processing workflow (Reinecke et al., 2012) and was log 2 transformed before statistical analysis. The Shapiro-Wilks test was used to test for normality, after which the Spearman Rank Correlation analysis was performed in IBM SPSS Statistics (v26).

### Statistical Analysis

Data analysis was conducted in Excel (2019) and IBM SPSS Statistics (v26). Scatter plots were generated in Spyder (Python 3.7) (Raybaut, 2009) using the matplotlib package (Hunter, 2007), REL: v3.4.2 (Caswell et al., 2021). All tests with *p* value less than 0.05 were considered statistically significant. For detailed statistical analyzes for each dataset, see the relevant sections in “Materials and Methods” and “Results.”

## Results

### Autism Spectrum Disorder Cohort Phenotype and Demography

The individuals in our study with ASD spanned the full range of developmental phenotypes observed in ASD. This is reflected in both the number of different Autism Diagnostic Observation Schedule, Second Edition (ADOS-2) Modules used for assessments and the autism severity scores (Supplementary Table 1). Each ADOS-2 Module is tailored to match different developmental levels, ranging from pre-verbal individuals with ASD to those with fluent speech. Although our cohort comprised of different demographic groups, demography did not correlate with any ASD trait or any molecular marker (data not shown).

### PGC-1α Promoter Is Hypermethylated in ASD

**FIGURE 1:**
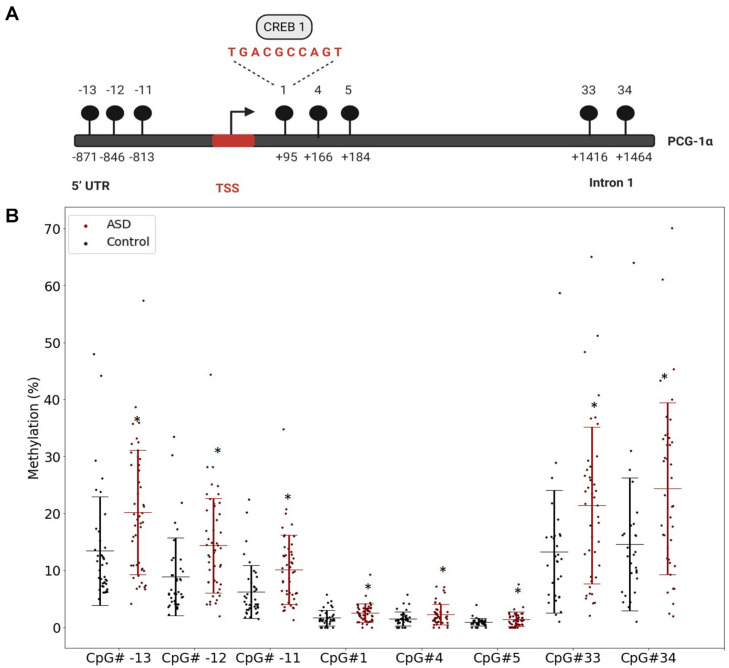
The PGC-1α promoter is hypermethylated in autism spectrum disorder (ASD). **(A)** Diagrammatic representation of the *PCG-1*α gene promoter region (Chr4:23889974) showing the location of the eight hypermethylated CpG sites (black circles) relative to the transcription start site (TSS) and the sequence of the binding site for the transcription factor, CREB1. **(B)** Percentage methylation of the eight differentially methylated *PGC-1*α promoter CpG sites measured using Targeted Next-Generation Bisulfite Sequencing (ASD *n* = 55, controls *n* = 44) (UTR = untranslated region). Differential methylation was identified using a two-tailed unpaired *t*-test with unequal variance (*p* < 0.05). Data represent the percent methylation in each individual at each site; box plots represent the mean percent methylation in either controls (black) or ASD (red) at each site; error bars represent standard deviations.

Our previous study implicated mitochondrial dysfunction in our South African ASD cohort, therefore we investigated whether DNA methylation contributed specifically to the regulation of mitochondrial biogenesis by measuring the methylation of *PGC-1*α, a central transcriptional regulator of mitochondrial biogenesis, in a larger sample of participants. We defined highly variable CpGs as those sites where the methylation range exceeded 5% across all samples to identify functionally significant DM genes. This threshold is consistent with *in vitro* (Ziller et al., 2013) and *in vivo* (D’Aquila et al., 2020) methylation studies. There were 12 highly variable CpG sites in *PGC-1*α that were significantly DM (*p* < 0.05) between ASD and controls (ASD *n* = 55, controls *n* = 43) (Supplementary Table 2). Of these, eight CpG sites were hypermethylated in ASD and clustered around the transcriptional start site (TSS), between the 5′ untranslated region (UTR) and intron 1 (Figures 1A,B), while four sites, located at intron 2, intron 12, and the 3′UTR were hypomethylated in ASD (Supplementary Figure 1).

**FIGURE 2:**
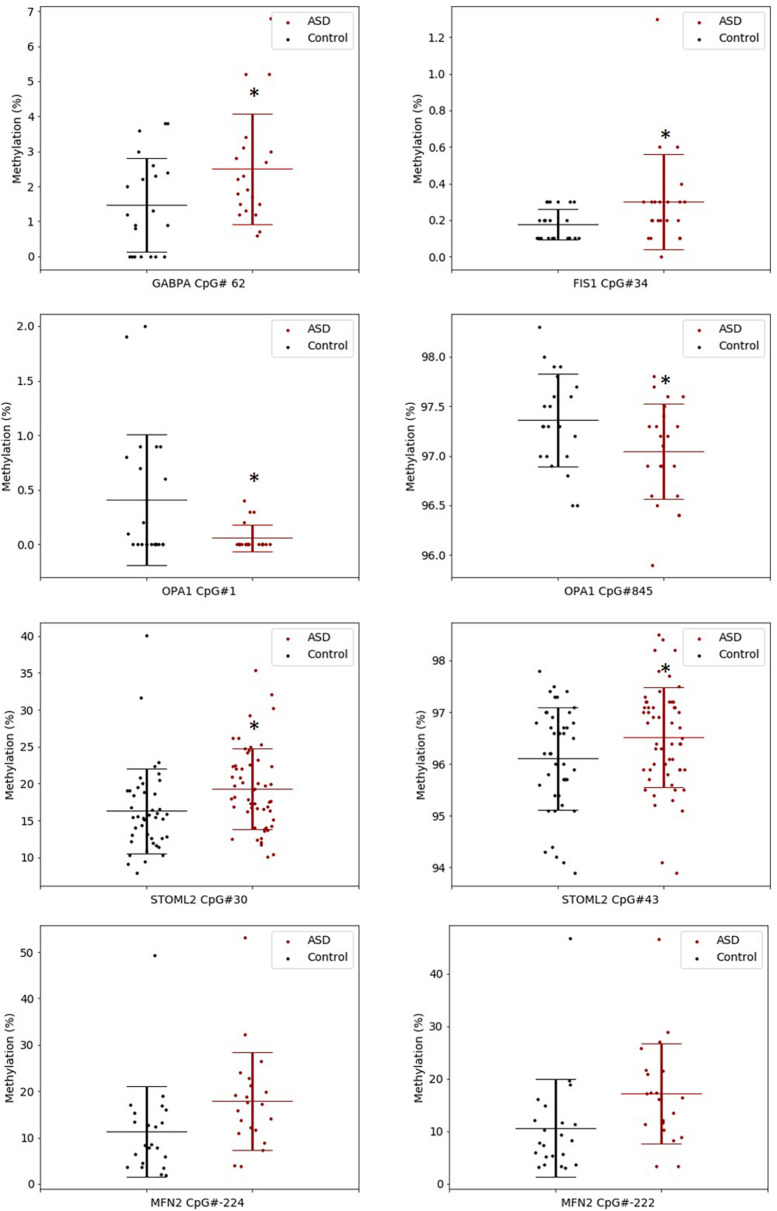
Key gene regulators of mitochondrial biogenesis, fission and fusion are differentially methylated in ASD. Box plots show the percentage methylation of the differentially methylated CpG sites (*p* < 0.05), measured using Targeted Next-Generation Bisulfite Sequencing of *STOML2* (ASD *n* = 55, controls 44) and *FIS*, *MFN2*, *OPA1*, and *GABPA* (ASD *n* = 22, controls *n* = 22). Differential methylation was identified using a two-tailed unpaired *t*-test with unequal variance (*p* < 0.05). Data represent the percent methylation in each individual at each site; box plots represent the mean percent methylation across either controls (black) or ASD (red) at each site; error bars represent standard deviations.

**FIGURE 3:**
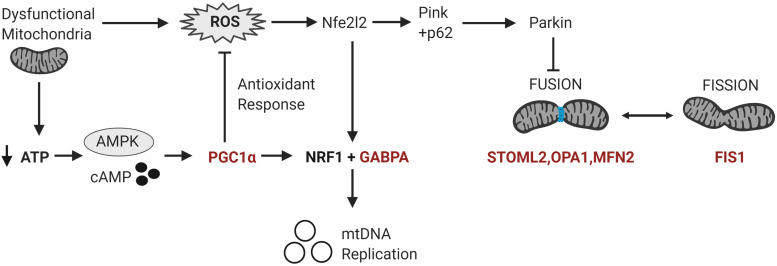
Differentially methylated genes converge on pathways regulating mitochondrial homeostasis in response to metabolic and oxidative stress. Mitochondrial homeostasis is maintained by differentially methylated genes (in red) involved in mitochondrial biogenesis, fission and fusion that converge on the regulation of mitochondrial copy number in response to metabolic and oxidative stress. Metabolic stress, which decreases ATP production, activates cAMP and AMPK signaling leading to the transcription and activation of *PGC-1*α. *PGC-1*α upregulates the expression of *Nrf1* and *GABPA* which induce the transcription of the mitochondrial transcription factors to facilitate mitochondrial biogenesis. *PGC-1*α also upregulates the transcription of antioxidant enzymes to modulate oxidative stress or ROS levels. Oxidative stress activates the redox-sensitive *Nfe2l2* pathway, which upregulates both *GABPA* and the Pink-Parkin pathway that controls mitophagy, fission and fusion (Nfe2l2 = nuclear factor erythroid 2-related factor 2; NRF1 = Nuclear respiratory factor 1; p62 = ubiquitin-binding protein p62; Pink = PTEN-induced kinase 1; ROS = reactive oxygen species).

The significant DM sites of *PGC-1*α in our study are consistent with a role for DNA methylation in regulating mitochondrial biogenesis. To examine this hypothesis, we quantified DNA methylation of *GABPA*, the transcriptional regulator of mitochondrial biogenesis that acts directly downstream of *PGC-1*α, as well as four genes involved in mitochondrial fission and fusion (*STOML2, MFN2, OPA1*, and *FIS1*) in a subset of our cohort (*n* = 22 ASD, *n* = 22 controls). *STOML2* contained two DM CpG sites, located in intron 2 and exon 5 downstream of the TSS; these sites were hypermethylated in ASD (Figure 2 and Supplementary Table 2). Significant DM sites between ASD and controls were also identified at one CpG site in *GABPA* and *FIS1*, and at two sites in *MFN2* and *OPA1* (Figure 2 and Supplementary Table 2). Therefore, we observed multiple DM mitochondrial biogenesis, fission and fusion genes converging on the regulation of mitochondrial homeostasis in ASD (Figure 3), congruent with a role for DNA methylation in the dysregulation of mitochondrial function in our cohort.

### PGC-1α Hypermethylation Is Associated With Altered Mitochondrial Function

We examined whether methylation affected mitochondrial biogenesis and function because DNA methylation was altered at several key regulators of mitochondrial biogenesis in our ASD cohort. *In silico* transcription factor binding site analysis of DM CpG sites in the *PGC-1*α promoter predicted a putative binding site for the transcription factor CAMP response binding element 1 (CREB1) at the CpG#1site (*p* = 1 × 10^–6^). This suggests that the DM site in ASD may have functional significance, which we examined by assessing mitochondrial function in our cohort. This was done by quantifying mtDNA copy number and deletions, as well as the levels of urinary metabolites typically associated with mitochondrial disease in ASD relative to controls.

### Mitochondrial DNA Copy Number Is Increased in ASD

**FIGURE 4:**
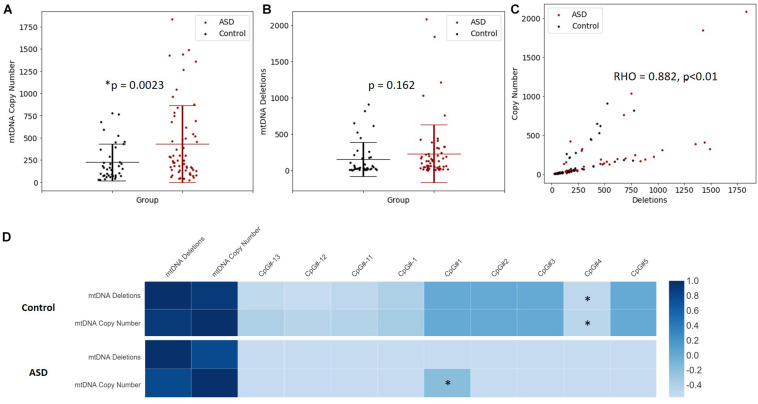
Relationship between mitochondrial DNA (mtDNA) copy number, mtDNA deletion and *PGC-1*α promoter methylation. **(A)** mtDNA copy number is significantly elevated in ASD. Relative quantification of mtDNA copy number was performed by multiplex real-time qPCR of *MT-ND1* and *B2M* (ASD *n* = 59, controls *n* = 40). **(B)** A non-significant increase in mtDNA deletions is observed in ASD. Relative quantification of mtDNA deletion was performed by multiplex real-time qPCR of *MT-ND1* and *MT-ND4* (ASD *n* = 59, controls *n* = 40). Significance was established using Student *t*-tests where (*p* < 0.05). **(C)** mtDNA copy number correlates significantly with mtDNA deletion in ASD and controls (*n* = 99), where Spearman’s rho = 0.882, *p* < 0.001. **(D)** The relationship between *PGC-1*α promoter methylation, mtDNA copy number and mtDNA deletions is altered in ASD. Heatmap shows Spearman rank correlations between mtDNA deletions, mtDNA copy number and percentage methylation at differentially methylated CpG sites in the *PGC-1*α promoter (ASD *n* = 49, control *n* = 42). Spearman’s rho is represented according to the color key provided, ^∗^ indicates *p* < 0.05. *PGC-1*α CpG#1 correlated positively with mtDNA copy number in ASD (Spearman’s *r* = 0.9, *p* = 8.814 10-10) while CpG#4 correlated negatively with both mtDNA copy number and (Spearman’s *r* = –0.4, *p* = 0.045) and mtDNA deletions (Spearman’s *r* = –0.4, *p* = 0.032) in controls.

Increased mtDNA copy number is a compensatory response to mild oxidative stress (Gaziev et al., 2014; Santos et al., 2014) and is an established biomarker of mitochondrial function (Castellani et al., 2020). mtDNA copy number of *MT-ND1* relative to *B2M* was significantly elevated in the ASD compared to the controls (*p* = 0.002) (Figure 4A). Mitochondrial deletions are typical of mitochondrial disease, therefore we examined whether mitochondrial deletions differed in ASD compared to controls by quantifying the copy number of the mitochondrial gene *MT-ND1* relative to *MT-ND4*; the latter resides in the major mitochondrial deletion arc. While mtDNA deletions were not significantly elevated in ASD relative to controls (*p* = 0.162), we observed markedly elevated mtDNA deletions in some ASD individuals (Figure 4B). Notably, mtDNA copy number correlated significantly (Spearman’s *r* = 0.9, *n* = 49, *p* = 8.814 × 10^–10^) with mtDNA deletions (Figure 4C), which suggests that elevated mtDNA copy number is associated with mitochondrial dysfunction in our cohort.

### PGC-1α Promoter Methylation Correlates Significantly With mtDNA Copy Number and This Relationship Is Altered in ASD

We examined whether *PGC-1*α promoter methylation is associated with mtDNA copy number and/or deletions, and thus mitochondrial function in ASD. We observed a significant correlation between DNA methylation at the *PGC-1*α promoter and mtDNA copy number, and this relationship differed between ASD and control groups. In the control group, *PGC-1*α methylation at CpG#4 correlated negatively with both mtDNA copy number (Spearman’s *r* = −0.4, *n* = 42, *p* = 0.045) and mtDNA deletions (Spearman’s *r* = −0.4, *n* = 42, *p* = 0.032; Figure 4D). However, in the ASD group, there was a significant positive correlation between *PGC-1*α methylation at CpG#1 and mtDNA copy number (Spearman’s *r* = 0.9, *n* = 49, *p* = 0.04; Figure 4D), with no correlation between *PGC-1*α methylation and mtDNA deletions. This suggests that *PGC-1*α methylation is associated with mitochondrial biogenesis and function and that this relationship is disrupted in our ASD group.

### DNA Methylation and mtDNA Copy Number Are Associated With Metabolomic Markers of Mitochondrial Dysfunction

**FIGURE 5:**
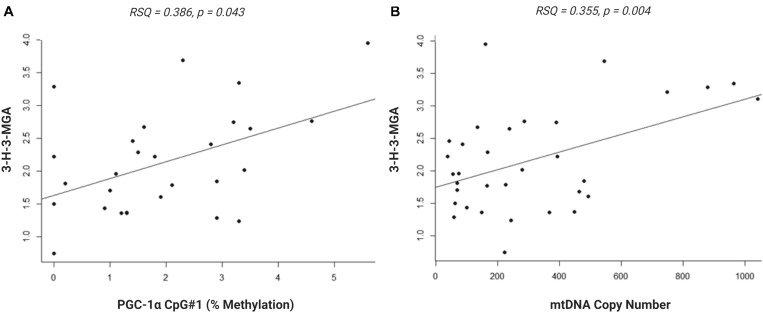
Urinary metabolite, 3-hydroxy-3-methylglutaric acid (3-H-3-MGA), correlates with PGC-1α methylation and mitochondrial DNA (mtDNA) copy number. Normal linear regression analysis shows that **(A)** DNA methylation at *PGC-1*α CpG#1 correlates with 3-H-3-MGA levels, RSQ = 0,2163, Spearman Rho = 0.386, *p* = 0,043 and **(B)** mtDNA copy number correlates with 3-H-3-MGA, RSQ = 0,2282, Spearman Rho = 0.355, *p* = 0,004; (ASD *n* = 20, control *n* = 13).

**FIGURE 6:**
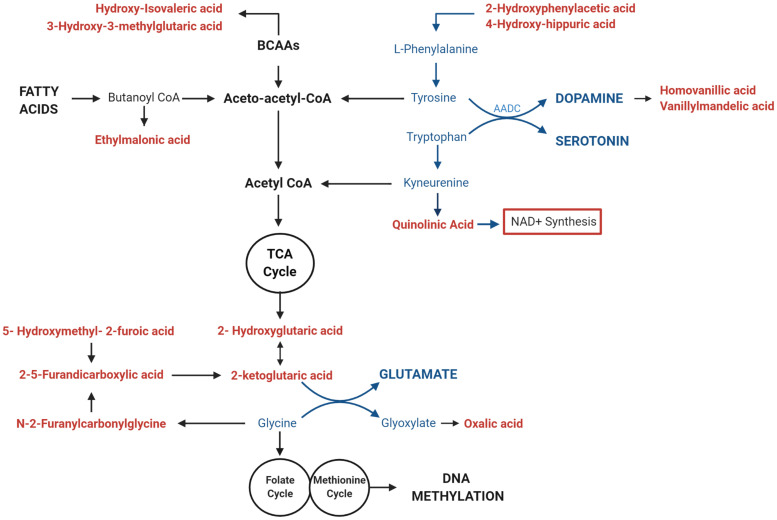
Mitochondrial DNA (mtDNA) copy number correlates with urinary metabolites implicating mitochondrial metabolism and neurotransmitter synthesis. Urinary metabolites that most significantly correlate with mtDNA copy number (*p* < 0.01) converge on pathways involved in the metabolism of fatty acids (ethylmalonic acid); Branched Chain Amino Acids (BCAAS) (hydroxy-isovaleric acid, 3-hydroxy-3-methylglutaric acid); and neurotransmitters including Dopamine (2-hydroxyphenylacetic acid, 4-hydroxyhippuric acid, vanillylmandelic acid, and homovanillic acid), Serotonin (Quinolinic acid), Glutamate (2-ketoglutaric acid, D-L-hydroxyglutaric acid; 2,5-furandicarboxylic acid, hydroxymethyl-2-furoic acid), and Glycine (*N*-2Furanylcarbonylglycine, oxalic acid). Metabolites that are associated with elevated mtDNA copy number are shown in red; pathways involved in neurotransmitter metabolism are highlighted in blue.

We investigated whether the differential methylation of *PGC-1*α and elevated mtDNA copy number observed in ASD were associated with metabolomic evidence of mitochondrial dysfunction. We examined the correlation between *PGC-1*α methylation, mtDNA copy number and levels of urinary organic acids which had previously been measured (ASD *n* = 20, controls *n* = 13) using GC-MS (Stathopoulos et al., 2020). DNA methylation at *PGC-1*α CpG#1, which is DM in our samples, correlated significantly with three of the 55 urinary organic acids tested (Supplementary Table 3B). MtDNA copy number correlated with 22 urinary metabolites associated with mitochondrial dysfunction (Supplementary Table 3A). Notably, both *PGC-1*α methylation and mtDNA copy number are associated with metabolites derived from Branched-chain amino acid (BCAA) metabolism, including 3-hydroxy-3-methylglutaric acid (3-H-3-MGA), which correlated significantly with both *PGC-1*α CpG#1 (*p* = 0.043) and with mtDNA copy number (*p* = 0.005) (Figures 5A,B). In addition, mtDNA copy number is associated most significantly (*r* < −0.3 or >0.3; *p* < 0.01) with metabolites derived from four metabolic pathways: fatty acid oxidation, phenylalanine-dopamine synthesis pathway, glycine-glutamine metabolism, and BCAAs (Supplementary Table 3A). These pathways converged on mitochondrial OXPHOS, one-carbon metabolism and neurotransmitter synthesis (Figure 6). Our data are consistent with an established metabolomic model for altered mitochondrial metabolism and neuroendocrinology (Yu et al., 2016) and supports an association between DNA methylation, elevated mtDNA copy number and mitochondrial dysfunction in ASD.

## Discussion

Autism spectrum disorder is a heritable, complex phenotype with numerous molecular pathways contributing to its etiology (Sandin et al., 2017). Despite the high heritability of ASD, there is no single or simple genetic mutation that accounts for ASD, and the disorder is characterized by phenotypic and clinical heterogeneity. This implies that epigenetic mechanisms may be important in ASD, and DNA methylation is known to contribute to ASD etiology (Wiśniowiecka-Kowalnik and Nowakowska, 2019). DNA methylation dysregulates many biological pathways in ASD, including, but not limited to, immune function (Nardone et al., 2017), chromatin remodeling (Andrews et al., 2018), synaptic signaling and neuronal regulation (Wong et al., 2019). Increasingly, mitochondrial biological pathways have also been implicated in ASD (Oliveira et al., 2005; Dhillon et al., 2011; Tang et al., 2013). The link between mitochondrial dysfunction and ASD is unsurprising given that efficient ATP production is essential for brain development and function. However, the relationship between DNA methylation and mitochondrial function is not fully understood.

Our data examined this relationship by testing the hypothesis that mitochondrial biogenesis and fusion genes are DM between ASD and controls and that this differential methylation affects mitochondrial function in ASD. Previously, we performed a whole-epigenome DNA methylation screen which identified 898 DM genes in a cohort of South African children (*n* = 48) which converged on nine canonical pathways involved in mitochondrial metabolism (Stathopoulos et al., 2020). Targeted next-generation bisulfite sequencing of a subset of genes and DNA pyrosequencing of two DM genes confirmed the DM observed in the whole-epigenome screen. Urinary metabolomic data in a subset of this cohort supported our hypothesis of mitochondrial dysfunction in ASD. Given this association between DM and general mitochondrial metabolism (Stathopoulos et al., 2020), this paper focused on the role of DNA methylation in regulating mitochondrial biogenesis in a larger South African ASD cohort by examining the link between *PGC-1*α methylation, mtDNA copy number and metabolomic evidence of mitochondrial dysfunction.

The transcriptional regulator of biogenesis, *PGC-1*α, was significantly DM between ASD and controls in our cohort, with the promoter region being hypermethylated in ASD. The promoter region included a CpG site (CpG#1) containing a putative transcription binding site for CREB1, which is a potent activator of *PGC-1*α transcription (Wu et al., 2006). Although we were not able to directly examine *PGC-1*α transcription in our cohort, this CREB1 site is reported to be DM in metabolic disease (Sookoian et al., 2010) which suggests that DM sites at the *PGC-1*α promoter region and TSS in our ASD cohort could affect gene transcription and subsequently, mitochondrial biogenesis and function.

Mitochondria are adaptive to changing cellular metabolic demands, thus they are dynamic organelles that are remodeled by biogenesis, fission, and fusion (Wai and Langer, 2016). Therefore, we measured the methylation of additional genes involved in mitochondrial biogenesis, fission and fusion and found that *GABPA*, which facilitates mtDNA replication downstream of *PGC-1*α in ASD, was DM (Ploumi et al., 2017). We also found that genes involved in mitochondrial fission (*STOML2, MFN2*, and *OPA1*) and fusion (*FIS1*) were DM in ASD. Mitochondrial fusion is induced in response to ROS stress to maintain optimal mitochondrial function and homeostasis (Karbowski and Youle, 2003). Collectively, these DM genes converge on the pathways regulating mitochondrial homeostasis in response to metabolic and oxidative stress (Figure 3). Of note, *STOML2* was hypermethylated at two CpG sites downstream of the TSS in ASD and it was the most significantly DM gene in our ASD cohort in our earlier study (Stathopoulos et al., 2020). The STOML2 protein plays an important role in mitochondrial fusion by stabilizing the OPA1 protein, which facilitates fusion of the inner mitochondrial membranes (Duvezin-Caubet et al., 2006; Ishihara et al., 2006; Tondera et al., 2009). STOML2-deficient cells fail to undergo mitochondrial fusion during stress, leading to mitochondrial fragmentation (Tondera et al., 2009). *STOML2* is also well-established as an anti-apoptotic gene in cancer cells, highlighting the importance of fusion to re-establish mitochondrial homeostasis and prevent mitophagy (autophagy of mitochondria) under stress. Our data is consistent with previous work showing that both fission and fusion genes are differentially expressed in ASD (Tang et al., 2013; Carrasco et al., 2019; Pecorelli et al., 2020). This supports the link between ASD and mitochondrial fusion and fission which highlights the differential methylation of mitochondrial genes on an integrated pathway level.

Our data show genes that regulate mitochondrial biogenesis, fission and fusion are DM in our cohort. The relationship between DM and mRNA expression is complex; hyper- or hypo-methylation can either increase or decrease gene expression depending on the gene as well as where in the gene region the methylation occurs (Arechederra et al., 2018; Tremblay and Jiang, 2019; Rauluseviciute et al., 2020). The low integrity of RNA extracted from the buccal cells used in our study meant we were not able to determine how the DM we observed impacted gene expression in our cohort. However, previous studies have shown that DM alters the expression of these genes (Tang et al., 2013; Carrasco et al., 2019; Pecorelli et al., 2020; Yang et al., 2020). Additionally, we investigated whether mtDNA copy number, a marker of mitochondrial function (Castellani et al., 2020), was altered in our cohort. Changes in mtDNA copy number have been reported in ASD, with both increases (Gu et al., 2013; Chen et al., 2015; Yoo et al., 2017) and decreases (Valiente-Pallejà et al., 2018; Singh et al., 2020) observed in ASD. These discrepancies can be attributed to several factors that differed across studies, including the age of the participants studied, the presence of co-morbidities and the degree of mitochondrial dysfunction in participants. We observed a significant increase in mtDNA copy number in our ASD group compared to controls, which represents an established compensatory mechanism in response to mitochondrial dysfunction (Ventura-Clapier et al., 2008). We also observed a significant positive correlation of mtDNA copy number with mtDNA deletions in ASD, suggesting that elevated mtDNA copy number is indicative of mitochondrial dysfunction in our cohort. Increased mtDNA copy number is observed as a response to oxidative stress in animal models and *in vitro* studies (Al-Kafaji and Golbahar, 2013; Masser et al., 2016; Zeng et al., 2019), as well as in human clinical studies using buccal samples (Kolbasina et al., 2020). This compensatory mechanism has also been reported in mitochondrial diseases (Bai et al., 2004; Grady et al., 2014; Thompson et al., 2016), neuropsychiatric and neurodevelopmental disorders (Montier et al., 2009; Giulivi et al., 2010; Chung et al., 2019; Chen et al., 2020; Ridout et al., 2020).

A correlation between *PGC-1*α methylation and mtDNA copy number is established with numerous studies reporting that *PGC-1*α promoter methylation is consistently associated with reduced mRNA expression and lower mtDNA copy numbers in a range of different tissues (Lillycrop et al., 2005; Ling et al., 2008; Barrès et al., 2009; Sookoian et al., 2010; Chen et al., 2012; Gillberg et al., 2014; Heinonen et al., 2015; Kelstrup et al., 2016; Kresovich et al., 2018; Liu et al., 2019; Petrie et al., 2020; Yang et al., 2020). These include studies that focused on both neurological (Yang et al., 2020) and metabolic disorders (Sookoian et al., 2010; Zhao et al., 2017; Kresovich et al., 2018). Consistent with this, we report a negative correlation between *PGC-1*α promoter hypermethylation and both mtDNA copy number and deletion in our control group. Thus, hypomethylation of the *PGC-1*α promoter is associated with increased mtDNA copy number and deletion. This is consistent with hypomethylation leading to an upregulation of PGC-1α transcription, and subsequently, *PGC-1*α-dependent mitochondrial biogenesis under conditions of mild mitochondrial dysfunction.

However, this relationship between *PGC-1*α promoter methylation and mtDNA copy number was perturbed in ASD. We observed a significant positive correlation between *PGC-1*α promoter methylation at CpG#1 and mtDNA copy number. This is consistent with observed *PGC-1*α promoter methylation being associated with the mtDNA copy number alterations seen in our ASD cohort. However, given that *PGC-1*α methylation is associated with reduced mRNA expression, this does not necessarily mean that *PGC-1*α methylation directly alters mtDNA copy number in ASD by dysregulating mitogenesis. Instead, it shows that hypermethylation of *PGC-1*α is associated with a dysregulation of mitochondrial homeostasis in our ASD cohort. This is consistent with the systemic metabolic dysfunction associated with ASD, and the central role played by *PGC-1*α in regulating mitochondrial metabolism and homeostasis. Hypermethylation at the *PGC-1*α promoter could inhibit the *PGC-1*α-dependent activation of antioxidant genes (St-Pierre et al., 2006) which is congruent with the evidence for elevated oxidative stress in ASD (Bjørklund et al., 2020). Oxidative stress can induce mitochondrial biogenesis via nuclear factor erythroid 2-related factor 2 (*Nfe2l2*) (Erlank et al., 2011) which upregulates *GABPA* independently of *PGC-1*α in a redox-sensitive manner (Kolbasina et al., 2020). We found no relationship between *PGC-1*α methylation and mtDNA deletion in ASD, suggesting an absence of adaptive hypomethylation to compensate for metabolic and putative oxidative stress.

To further explore the link between differential methylation, mtDNA copy number and mitochondrial function, we used metabolomics analysis, which directly reflects the biochemical activity, including mitochondrial activity, of a biological sample (Glinton and Elsea, 2019). Our previous study examined which metabolites were significantly altered in ASD compared to controls, using a panel of urinary organic acids that are indicative of mitochondrial disease in South African children (Reinecke et al., 2012). Here, we used this metabolomic data in conjunction with the new data generated in this study to investigate whether *PGC-1*α methylation or mtDNA copy number correlated with metabolomic evidence of mitochondrial dysfunction in our cohort (Stathopoulos et al., 2020).

We found that mtDNA copy number was associated with a metabolomic profile that was consistent with a link between DNA methylation, mitochondrial dysfunction, and neuropathology. Metabolites derived from tyrosine, tryptophan, and glycine significantly (*p* < 0.01) correlated with mtDNA copy number. These are precursors of dopamine, serotonin, melatonin, and glutamate synthesis, which are all important neurotransmitters implicated in ASD etiology (Marotta et al., 2020). The notable enrichment of metabolites derived from the dopamine pathway, which is closely tied to the cellular oxidation state (Ben-Shachar, 2020), is consistent with altered redox homeostasis in ASD. Moreover, glycine serves as the precursor to one-carbon metabolism (cysteine, methionine, and glutathione pathways). These are essential regulators of an oxidative state which have been implicated in the metabolomic profile associated with ASD severity in two independent cohorts (Smith et al., 2020; Needham et al., 2021) and have been identified as a link between DNA methylation and mitochondrial dysfunction (Maddocks et al., 2016; Shen et al., 2020). In addition, both *PGC-1*α methylation and mtDNA copy number correlate significantly with metabolites derived from BCAA catabolism. BCAAs provide nitrogen to the glutamate-glutamine cycle and have been implicated as important regulators of glutamatergic neurotransmission (Yudkoff, 2017), which contribute to ASD etiology (Essa et al., 2013). Two metabolites [3-H-3-MGA and 3-methylglutaconic acid (3-MGA)] were significantly elevated in our ASD cohort (Stathopoulos et al., 2020) and are characterized as urinary biomarkers of mitochondrial respiratory chain deficiencies (Reinecke et al., 2012; Smuts et al., 2013). Altered BCAA metabolism has also been linked to oxidative stress resulting from perturbed NAD^+^/NADH redox ratios (Esterhuizen et al., 2017). Of note, mtDNA copy number correlated significantly with the direct precursor of *de novo* NAD^+^ synthesis, QA (*p* = 0.008), which is a neurotoxin (Notarangelo and Pocivavsek, 2017) that is also implicated in ASD etiology (Savino et al., 2020). NAD^+^ has also been identified as a metabolic link between changes in mtDNA copy number, methionine metabolism and DNA methylation (Lozoya et al., 2018). Therefore, both *PGC-1*α methylation and mtDNA copy number are associated with metabolomic evidence of mitochondrial dysfunction, oxidative stress, and neuropathology. Together, our metabolic data are consistent with a dysregulation of the link between methionine metabolism, mitochondrial dysfunction, and neurotransmitter synthesis in ASD.

Our study is one of the few molecular studies from Sub-Saharan Africa to examine ASD in an understudied African population. We present the first report of an association between the methylation of genes involved in mitochondrial biogenesis and remodeling, and mitochondrial function in a South African ASD cohort. While our results are correlative and cannot establish causality, they contribute to and are supported by, a growing body of evidence that point to aberrant DNA methylation and mitochondrial dysfunction in ASD etiology. Our results highlight the value of epigenetic research in under-studied populations to highlight novel associations. The central role of DNA methylation in modulating mitochondrial function highlights the potential to explore existing mitochondrial medications as putative therapeutic interventions for ASD symptomatology.

## Data Availability Statement

The original contributions presented in the study are included in the article/Supplementary Material, further inquiries can be directed to the corresponding author.

## Ethics Statement

The studies involving human participants were reviewed and approved by University of Cape Town, FSREC076-2014. Written informed consent to participate in this study was provided by the participants’ legal guardian/next of kin.

## Author Contributions

CO’R conceptualized the overall study design, was responsible for the phenotype data, supervised the laboratory work and data analysis, and was the major contributor in writing the manuscript. SB assisted with the design, data acquisition, and analysis of the mitochondrial DNA copy number data, and contributed to writing the manuscript. EB assisted with the design and analysis of the methylation data for the mitochondrial fission and fusion genes and contributed to writing the manuscript. CM assisted with the design and analysis of the methylation data for the mitochondrial biogenesis genes, analyzed the urinary metabolic data, and contributed to writing the manuscript. All authors read and approved the final manuscript.

## Conflict of Interest

The authors declare that the research was conducted in the absence of any commercial or financial relationships that could be construed as a potential conflict of interest.

## Publisher’s Note

All claims expressed in this article are solely those of the authors and do not necessarily represent those of their affiliated organizations, or those of the publisher, the editors and the reviewers. Any product that may be evaluated in this article, or claim that may be made by its manufacturer, is not guaranteed or endorsed by the publisher.

## Supplementary Material

Supplementary Figure 1 *PGC-1*α is differentially methylated in ASD. Targeted Next-Generation Bisulfite Sequencing (*n* = 55 ASD, *n* = 44 controls) identified differential methylation 12 CpG sites on *PGC-1*α. Differential methylation was identified using a two-tailed unpaired *t*-test with unequal variance (*p* < 0.05). Data represent the percent methylation in each individual at each site; box plots represent the mean percent methylation across either ASD or controls at each site; error bars represent standard deviations.

Supplementary Table 1 Summary of demographic and phenotypic characteristics of participants used in this study. ASD, autism spectrum disorder.

Supplementary Table 2 Differentially methylated CpG sites (*p* < 0.05, methylation range > 5%) of *PGC-1*α and *STOML2* (*n* = 55 ASD, 44 controls) and *GABPA*, *MFN2*, *FIS1*, and *OPA1* (*n* = 22 ASD, *n* = 22 controls).

Supplementary Table 3A Spearman correlation coefficients for urinary organic acids that correlate significantly with mtDNA copy number (*n* = 20 ASD, *n* = 13 controls). The most significant (*p* < 0.01) correlations with *r* < −0.3 or >0.3 are shown in bold, non-significant correlations are not shown. BCAA, branched chain amino acids; TCA, tricarboxylic acid cycle.

Supplementary Table 3B Spearman correlation coefficients for urinary organic acids (*n* = 20 ASD, *n* = 13 controls) that correlate significantly with methylation at promoter of *PGC-1*α at the CpG#1 site.

## Abbreviations

ADOS-2: Autism Diagnostic Observation Schedule Second Edition
ASD: autism spectrum disorder
B2M: Beta-2-microglobulin
BCAAs: Branched-chain amino acids
CREB1: CAMP response binding element 1
DM: Differentially methylated
FIS1: Mitochondrial fission protein 1
GABPA: GA binding protein transcription factor subunit alpha
mtDNA: Mitochondrial DNA
MFN2: Mitofusin 2
Nrf-2A: Nuclear respiratory factor 2 alpha subunit
ND1: Mitochondrially encoded NADH ubiquinone oxidoreductase core subunit 1
ND4: Mitochondrially encoded NADH ubiquinone oxidoreductase core subunit 4
OPA1: Optic atrophy 1
PGC-1 α: Peroxisome proliferator-activated receptor-gamma coactivator-1 alpha
STOML2: Stomatin-like protein 2
TSS: Transcriptional start site.
