# Supplementary figures and images for: DNA Methylation of PGC-1α Is Associated With Elevated mtDNA Copy Number and Altered Urinary Metabolites in Autism Spectrum Disorder
Source: Front Cell Dev Biol. 2021 Jul 26;9:696428. doi: 10.3389/fcell.2021.696428 (PMC8352569; doi:10.3389/fcell.2021.696428)

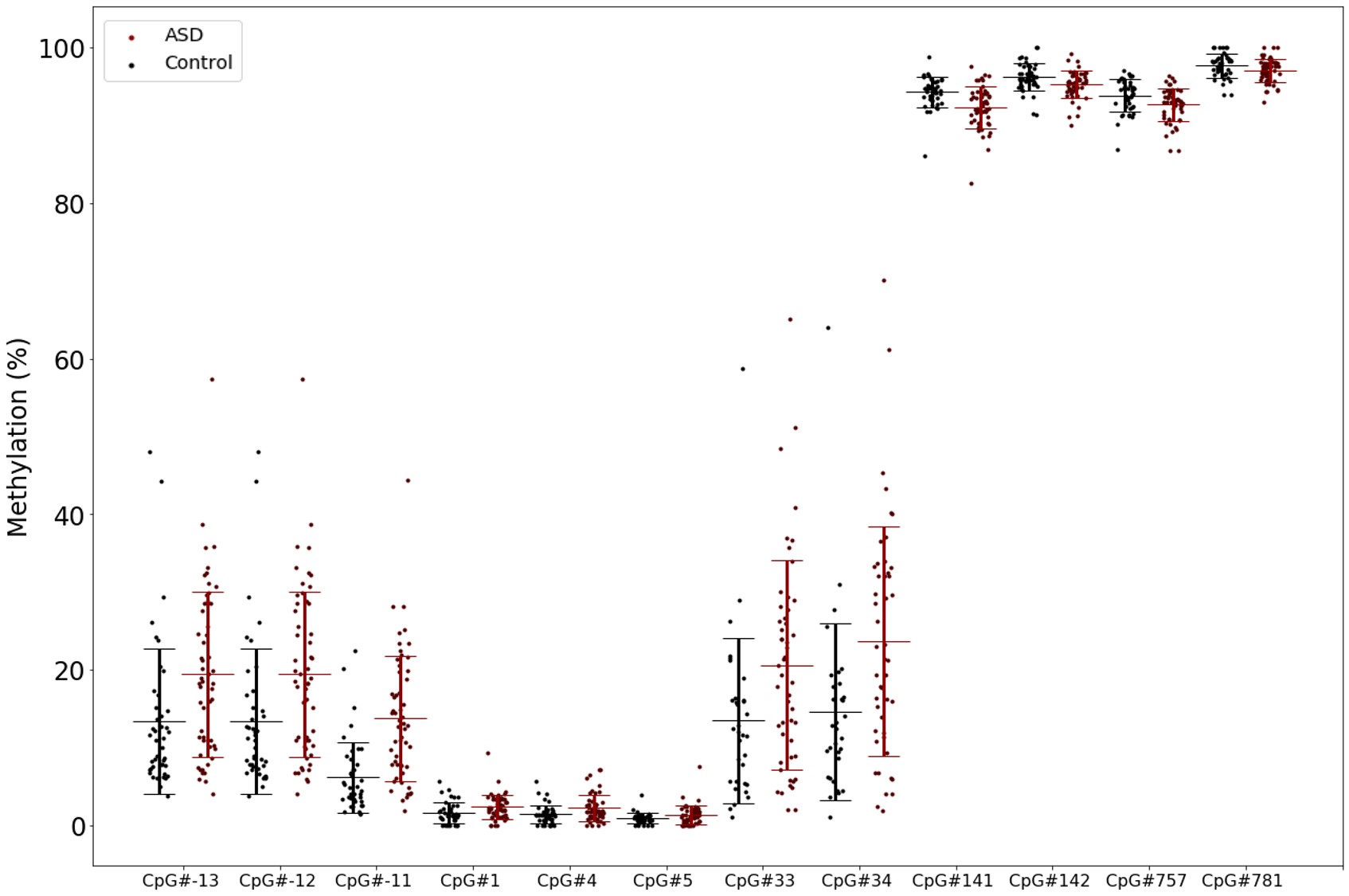

Supplement: Supplementary Figure 1 — PGC-1α is differentially methylated in ASD. Targeted Next-Generation Bisulfite Sequencing (n = 55 ASD, n = 44 controls) identified differential methylation 12 CpG sites on PGC-1α. Differential methylation was identified using a two-tailed unpaired t-test with unequal variance (p < 0.05). Data represent the percent methylation in each individual at each site; box plots represent the mean percent methylation across either ASD or controls at each site; error bars represent standard deviations. [file Image_1.jpg]
